# Tumor Location Is an Independent Prognostic Factor in Completely Resected Pathological Stage I Non-Small Cell Lung Cancer: A Multicenter Retrospective Study

**DOI:** 10.3390/cancers16091710

**Published:** 2024-04-27

**Authors:** Wei-Ke Kuo, Po-Ju Chen, Mei-Hsuan Wu, Hsin-Chung (Henry) Lee, Jiun-Kai Fan, Pang-Hung Hsu, Ching-Fu Weng

**Affiliations:** 1 Division of Respiratory Therapy and Chest Medicine, Department of Internal Medicine, Sijhih Cathay General Hospital, New Taipei 221, Taiwan; s1108108642@gmail.com; 2 Department of Bioscience and Biotechnology, National Taiwan Ocean University, Keelung 202, Taiwan; phsu@ntou.edu.tw; 3 Department of Thoracic Surgery, Sijhih Cathay General Hospital, New Taipei 221, Taiwan; cgh13499@cgh.org.tw; 4 Center of Teaching and Research, Hsinchu Cathay General Hospital, Hsinchu 300, Taiwan; s101082015@gapp.nthu.edu.tw; 5 Precision Medicine Ph.D. Program, National Tsing-Hua University, Hsinchu 300, Taiwan; 6 Department of Surgery, Cathay General Hospital, Taipei 106, Taiwan; hhc.lee@gmail.com; 7 Department of Diagnostic Radiology, Hsinchu Cathay General Hospital, Hsinchu 300, Taiwan; jkfan@cgh.org.tw; 8 Center of Excellence for the Oceans, National Taiwan Ocean University, Keelung 202, Taiwan; 9 Division of Pulmonary Medicine, Department of Internal Medicine, Hsinchu Cathay General Hospital, Hsinchu 300, Taiwan; 10 School of Medicine, National Tsing-Hua University, Hsinchu 300, Taiwan

**Keywords:** non-small cell lung cancer, early stage, lower lobe, survival, prognosis, histology

## Abstract

**Simple Summary:**

Early detection and curative-intent surgery are crucial for improving survival rates in patients with early-stage non-small cell lung cancer (NSCLC). However, even after complete tumor resection in patients with pathological stage I NSCLC, recurrence rates and overall survival remain unfavorable. There is growing evidence suggesting that the location of the primary tumor, nodal upstaging, certain genetic mutations, and histological characteristics have been found to be more prevalent in tumors located in specific lung regions. To address this research gap, we conducted a multicenter retrospective study in Taiwan to explore the independent prognostic significance of tumor location in patients with completely resected pathological stage I NSCLC. The results suggest that patients with NSCLC located in the lower lobe, especially those that are histologically classified as IASLC Grade 3, may require additional attention and interventions to improve outcomes. These findings have implications for personalized treatment strategies and the identification of high-risk patients who may benefit from closer surveillance or adjuvant therapies.

**Abstract:**

Previous studies suggested that the location of the primary tumor in non-small cell lung cancer (NSCLC) is associated with clinical features and prognosis, but results are conflicting. The purpose of this study was to explore tumor location as an independent risk factor of survival for patients with completely resected pathological stage I NSCLC. This was a multicenter retrospective study conducted in Taiwan. Included patients were diagnosed with stage I NSCLC and had undergone primary tumor resection. Variables including tumor location, pathological stage, histological differentiation, and International Association for the Study of Lung Cancer (IASLC) grade were evaluated for predictive ability for disease-free survival (DFS) and overall survival (OS). A total of 208 patients were included, with 123 (59.1%) patients having a primary tumor in the upper and middle lobes. The median duration of follow-up for survivors was 60.5 months. Compared to patients with IASLC Grade 3 disease, patients with Grade 1 disease had significantly longer DFS. Tumor location and IASLC grade were independent predictors for OS in multivariate analysis. Specifically, patients with NSCLC in the lower lobe and patients who are histologically classified as IASLC Grade 3 may have poorer prognosis and require greater attention to improve outcomes.

## 1. Introduction

Lung cancer is the leading cause of cancer death worldwide, with most patients having advanced disease at the time of diagnosis [1]. The most common type of lung cancer is non-small cell lung cancer (NSCLC), and although much progress has been achieved in the treatment of NSCLS, the mortality rate remains high [2]. Early diagnosis and curative-intent surgery are critical for improving the survival of patients with NSCLS. The development of low-dose computed tomography (LDCT) has increased the detection of early-stage lung cancer and allowed an increasing number of patients to undergo potentially curative complete resection [3,4]. Despite this advancement, many patients with pathological stage I NSCLC experience recurrence after surgery, and the 5-year post-lobectomy survival rate for these patients remains unfavorable at 45% to 65% [5].

Some evidence suggests that the site of primary NSCLC is associated with the clinical features and prognosis. The rate of nodal upstaging has been reported to be higher in NSCLC in the lower lobe compared to other lobes, which may be due to a greater likelihood of unsuspected nodal involvement [6]. Higher rates of positive epidermal growth factor receptor mutation and adenocarcinoma, especially invasive adenocarcinoma, have been reported in patients with cancer in the upper lung regions compared to other locations [7,8]. Findings regarding the association between the location of NSCLC and survival have been inconsistent. In a population-based cancer registration study including 33,919 mostly advanced-stage lung cancer patients in Taiwan, patients with lower lobe tumors had poorer survival compared to patients with tumors in the upper- and middle lobes [9]. A systematic review and meta-analysis conducted by Lee et al. revealed that stage I–III NSCLC located in the upper lobes was associated with higher 5-year survival rates compared to tumors in other locations [10]. A population-based analysis by Ou et al. found that stage IB NSCLC with a non-upper lobar tumor location carried an increased mortality risk [5]. In contrast, the results of several studies have indicated that the tumor location within the lung does not significantly predict survival in pathologic stage I and II NSCLC [11,12,13]. Due to the conflicting reports in the existing literature, the association between the location of NSCLC, especially stage I NSCLC, and prognosis warrants further investigation. The purpose of this study was to explore tumor location as an independent risk factor of prognosis for patients with completely resected pathological stage I NSCLC.

## 2. Materials and Methods

This was a multicenter, retrospective chart review study conducted in Taipei Cathay General Hospital, Sijhih Cathay General Hospital, and Hsinchu Cathay General Hospital in Taiwan. We evaluated all adult patients (≥18 years) diagnosed with pathology proven to be stage I NSCLC between January 2007 and December 2020. Included patients were pathologically diagnosed as stage I NSCLC according to the American Joint Committee on Cancer (AJCC) staging system (2007 to 2009: AJCC 6th edition, 2010 to 2017: AJCC 7th edition, 2018 to 2020: AJCC 8th edition) and had undergone primary tumor resection with curative intent and systemic mediastinal lymph node dissection. Patients who presented with synchronous primary lung cancer were excluded. This study was approved by the Institutional Review Board of Cathay General Hospital (CGH-P110076).

The following information was collected from the medical records of the included patients at the time of diagnosis: age, sex, comorbidities, smoking status, tumor location, histological differentiation (categorized as well, moderately, and poorly differentiated), tumor grade according to the International Association for the Study of Lung Cancer (IASLC) grading system (categorized as Grade 1, Grade 2, or Grade 3) [14], lymphocyte percentage, neutrophil percentage, and neutrophil-to-lymphocyte ratio.

Data were analyzed using the Statistical Package for Social Sciences, version 22 (SPSS Software, Chicago, IL, USA). Differences between groups were assessed using independent T-test or chi-square test. Disease free survival (DFS) was defined as the time from initial surgery to recurrence of disease or death, and overall survival (OS) was defined as the time from initial surgery to death or last follow-up. To determine independent predictors of survival outcome, we used the Cox proportional hazard models with backward stepwise analysis. Variables with *p* value < 0.05 in univariate analysis were entered into multivariate analysis. Hazard ratios (HRs) and 95% confidence intervals (CIs) were calculated, and Kaplan–Meier analysis with a log-rank test was used to compare time-dependent outcomes. A *p* value of <0.05 was considered significant.

## 3. Results

### 3.1. Demographic Data

The characteristics of the 208 included patients are summarized in Table 1. The location of the primary tumor was in the upper- or middle lobes in 123 (59.1%) patients and in the lower lobes in 85 (40.9%) patients. The mean age was 62 years, and most patients were female (61.5%). Grouped by primary tumor location (upper/middle lobes or lower lobes), there was no statistically significant difference between groups in terms of presence of comorbidities, smoking status, pathological stage, histological differentiation, IASLC grading, or immune cells.

### 3.2. Survival Analysis

**Figure 1:**
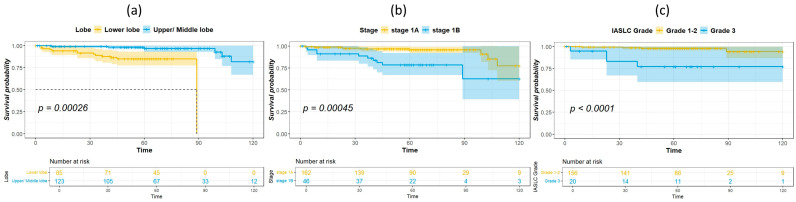
Kaplan–Meier survival curves for overall survival according to primary tumor location (lower lobe vs. upper/middle lobe) (**a**), pathological stage IA or IB (**b**), and IASLC Grade 1–2 vs. 3 (**c**).

**Figure 2:**
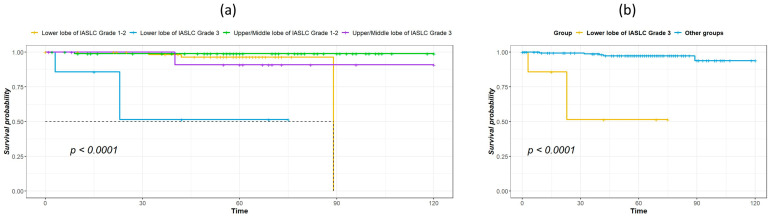
Kaplan–Meier survival curves for overall survival according to tumor location and IASLC histological grade: grouped by lower lobe or upper/middle lobe and by IASLC Grade 1/2 or Grade 3 (**a**), lower lobe of Grade 3 vs. combined lower lobe of Grades 1–2 and upper/middle lobe of Grades 1–3 (**b**).

**Figure 3:**
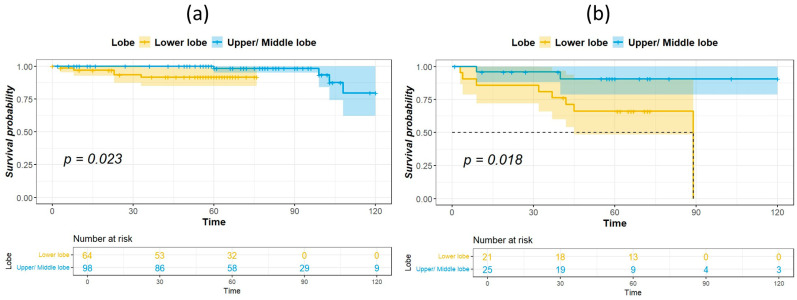
Kaplan–Meier survival curves for overall survival according to tumor location in patients with pathological stage IA (**a**) and IB (**b**) disease.

The median duration of follow-up for survivors was 60.5 months. Table 2 shows the results of univariate analysis of the predictor of DFS. Patients with IASLC Grade 1 disease had significantly longer DFS compared to patients with Grade 3 disease. The results of the univariate and multivariate analysis for predictors of OS are listed in Table 3. Age, tumor location, and IASLC grade were significant in univariate analysis and were entered into the multivariate model. In the multivariate analysis, tumor location and IASLC grade were independent predictors for OS. Kaplan–Meier curves for OS of patients with complete resection of stage I NSCLC according to primary tumor location, pathological stage, and IASLC grade are presented in Figure 1a–c. Patients with NSCLC in the upper- and middle lobes, pathological Stage IA, and IASLC Grade 1 and 2 had significantly longer OS compared with patients with primary tumor in the lower lobes, pathological Stage IB, and IASLC Grade 3 (log-rank = 15.31, 12.59, and 15.53, *p* < 0.001, <0.001, and <0.001, respectively). The OS of patients according to the combination of tumor location and IASLC grade is presented in Figure 2a,b. Patients with lower lobe tumors and IASLC Grade 3 disease had poorer OS compared with other groups (log-rank = 41.02, *p* < 0.001). Patients with tumors located in upper- and middle lobes had longer OS compared with those with tumors located in lower lobes in both pathological Stage IA (Figure 3a) and IB (Figure 3b) disease (log-rank = 7.35 and 5.58, *p* = 0.007 and 0.018, respectively).

## 4. Discussion

In this study, we demonstrated that the location of the primary tumor in pathological stage I NSCLC is an independent risk factor of survival in patients who have undergone complete tumor resection. In both stages IA and IB, the location of the primary tumor in the lower lobes was associated with poorer survival compared to the upper- and middle lobes. Furthermore, the IASLC grade was associated with the survival outcome, and patients with IASLC Grade 3 NSCLC occurring in the lower lobe had the poorest OS. Our study presents original findings that offer novel insights into the prognostic significance of tumor location in completely resected pathological stage I NSCLC.

Several theories as to why lower lobe tumors may carry a worse prognosis than upper lobe tumors in the lung have been proposed, and many theories attribute the difference in prognosis to clinical staging. Rocha et al. reported that approximately one-third of early-stage lung cancers were found to have been initially misclassified, and most cases were upstaged upon thoracotomy with lymph node dissection [6]. In that study, the location of primary tumor in the lower lobe was the only significant predictor of upstaging. In a study of 1513 patients with clinical stage I NSCLC who had undergone lobectomy (47% by video-assisted thoracoscopic surgery and 53% by thoracotomy), Licht et al. demonstrated that nodal upstaging was significantly more likely when the primary cancer was located in the lower lobe [15]. Difficulties in accurately determining the clinical staging of lower lobe tumors, especially those near the airway, pleura, and chest wall, may explain why lower lobe tumors are associated with a higher rate of upstaging [6,10,16]. However, since our study population was limited to patients with pathologically confirmed stage I NSCLC, the finding of inferior survival outcomes with lower lobe primary tumors compared to those located in the upper and middle lobes cannot be explained by incorrect staging.

Physiologically, the lower regions of the lungs receive a comparatively greater blood supply than the upper regions, and this relative overperfusion could potentially trigger more aggressive tumor behavior and contribute to higher occurrences of micrometastasis in patients with primary tumors located in the lower lobes [17]. In line with this theory, a study by Shaverdian et al. including patients with stage I NSCLC treated with stereotactic body radiation therapy found that compared to patients with a non-lower lobe primary tumor location, patients with lower lobe location had poorer 3-year relapse-free survival (75% vs. 89%, *p* = 0.04) and poorer 3-year OS (63% vs. 82%, *p* = 0.01) [18]. In our study, a lower lobe location was significantly associated with worse OS but not DFS, and these findings are consistent with those reported by Jeong et al. [19]. However, a poorer prognosis as a result of micrometastasis is likely inapplicable to patients with pathological stage I NSCLC who have undergone complete resection of the tumor and have received adjuvant chemotherapy as needed, such as those included in our study.

In a study of genomic alterations in lung squamous cell carcinoma by Okamoto et al., a significant correlation was observed between the tumor mutation burden (TMB) and tumor location, with cancers originating in the upper- or middle lobes exhibiting higher TMB compared to those originating in the lower lobes [20]. The upper lung regions have comparatively lower perfusion, which can lead to sluggish lymphatic drainage along the peribronchial system. This may result in increased exposure to harmful particles that can damage the lung epithelium, hence making these regions more susceptible to lung disease. Consequently, the accumulation of DNA damage caused by carcinogens may give rise to a higher frequency of genetic mutations in the upper lobe [21]. Interestingly, Devarakonda et al. reported that in patients who underwent resection for NSCLC, a high TMB was associated with more favorable survival outcomes compared to a low TMB [22]. Although a genetic study was not performed in this study, a lower TMB in the lower lobes may have contributed to a worse prognosis, and this theory warrants further study.

There may be other possible reasons explaining the findings of this study. Lee et al. revealed that patients who had lower lobe cancer exhibited a greater percentage of non-adenocarcinoma histology, higher levels of tumor markers, and a lesser proportion of epidermal growth factor receptor (EGFR) mutations, and a lower proportion of EGFR mutations was an independent risk factor for higher 5-year all-cause mortality [23]. Kanaji et al. demonstrated that the prognosis of NSCLC may be affected by the underlying chronic lung disease and its location. For instance, idiopathic pulmonary fibrosis is commonly found in the lower lobes and is correlated with a poorer prognosis of NSCLC [24]. Ueda et al. showed a significant relationship among the infectious complication, cancer recurrence, and lower lobe cancer in resected lung cancer [25]. Currently, data on whether the location of early-stage NSCLC tumor in the upper- or lower lung is related to prognosis are scarce and inconsistent, and mechanisms to explain differences in prognosis due to tumor location have not been definitively revealed. Large prospective studies and studies related to the tumor microenvironment are currently needed.

Another finding of our study is that the IASLC grade was associated with DFS and OS, and the findings are consistent with the study by Xu et al. on stage I lung adenocarcinoma [26]. Our study further evaluated the effect of tumor location and IASLC grade on prognosis, and our results show that patients with lower lobe tumor of IASLC Grade 3 had worse OS compared with lower lobe tumor of IASLC Grade 1–2, as well as upper/middle lobe tumors of IASLC Grade 1–2 or 3. Therefore, these patients may need more careful monitoring and further adjuvant treatment, such as immunotherapy or target therapy if suitable, to achieve better outcomes.

Furthermore, in lung adenocarcinoma, there can be differences in the distribution of stages based on gender. However, the exact gender distribution difference between stage IA and IB, specifically, may vary depending on various factors such as population demographics, geographic location, and underlying risk factors. In terms of the gender distribution within specific stages like IA and IB, there might not be a substantial difference. Both men and women can develop lung adenocarcinoma, and the distribution within stages may reflect broader trends in lung cancer incidence rather than specific differences between IA and IB stages. However, lung cancer limited to male and female patients may affect outcomes equally or differently, especially in the early stage; the need for further analysis of gender-specific factors, such as hormonal influences, genetic predispositions, and treatment responses, will be addressed more in future larger cohorts to prove this concept.

Finally, except for the discussion in this article, there is no direct mention of a relationship between the primary tumor location in the lung (upper/middle lobes or lower lobes) and smoking status in the existing literature. Our studies specifically investigating the correlation between tumor location and smoking habits in lung cancer patients could shed light on any potential associations or lack thereof.

This study has several limitations. First, the retrospective design of this study led to incomplete collection of data on factors related to prognosis, such as the type of surgical procedure, EGFR status, performance status, and pulmonary function. Second, we did not include covariates that could impact the post-progression survival outcome due to incomplete collection of data on factors related to prognosis, such as the performance status, adjuvant treatment, treatment after recurrence, and treatment strategy; these data may provide clues as to why the tumor location was associated with DFS but not OS. Third, we included patients between 2007 and 2020 in our study; however, we did not take into account the effect of advancements in surgical techniques and supportive care or different versions of the AJCC TNM staging system during the study period.

## 5. Conclusions

In conclusion, our study demonstrates that a lower lobe tumor location was an independent risk factor of survival in patients with pathological stage I NSCLC who had undergone complete resection. Specifically, patients with NSCLC located in the lower lobe and histologically classified as IASLC Grade 3 may have poorer prognosis and may require greater attention to improve outcomes.

## Figures and Tables

**Table 1:** Characteristics of patients according to location of primary lung tumors.

	Upper/Middle Lobe (*n* = 123)	Lower Lobe (*n* = 85)	*p* Value
Age (years)	62.8 ± 1.0	59.9 ± 1.3	0.083
Male	49 (39.8%)	31 (36.5%)	0.624
Comorbidities			
Hypertension	60 (50.4%)	30 (37.5%)	0.073
Diabetes mellitus	19 (16.0%)	8 (10.0%)	0.228
Asthma	7 (5.9%)	5 (6.3%)	1.000
COPD	2 (1.7%)	4 (5.0%)	0.222
CKD	6 (5.0%)	2 (2.5%)	0.479
Smoking status			0.279
Current or former	28 (22.8%)	25 (29.4%)	
Never	95 (77.2%)	60 (70.6%)	
Surgical extent			<0.001
Wedge resection	23 (18.7%)	16 (18.8%)	
Segmentectomy	28 (22.8%)	42 (49.4%)	
Lobectomy	71 (57.7%)	24 (28.2%)	
Adjuvant treatment			0.055
With	13 (10.6%)	17 (20%)	
Without	109 (88.6%)	67 (78.8%)	
Pathological stage			0.454
Stage IA	98 (79.7%)	64 (75.3%)	
Stage IB	25 (20.3%)	21 (24.7%)	
Histology			0.492
Adenocarcinoma	112 (91.1%)	73 (85.9%)	
Squamous cell carcinoma	6 (4.9%)	6 (7.1%)	
Others	5 (4.1%)	6 (7.1%)	
Histological differentiation			0.158
Well-differentiated	35 (33.3%)	30 (45.5%)	
Moderately differentiated	65 (61.9%)	31 (47.0%)	
Poorly differentiated	5 (4.8%)	5 (7.6%)	
IASLC grading			0.652
Grade 1	26 (24.3%)	21 (30.4%)	
Grade 2	68 (63.6%)	41 (59.4%)	
Grade 3	13 (12.1%)	7 (10.1%)	
Immune cells			0.652
Lymphocyte (%)	29.4 ± 0.9	30.1 ± 1.0	
Neutrophil (%)	60.9 ± 0.8	60.5 ± 1.2	
NLR	3.2 ± 0.6	2.5 ± 0.2	

Data are presented as mean ± standard deviation or number (%); COPD, chronic obstructive pulmonary disease; CKD, chronic kidney disease; IASLC, International Association for the Study of Lung Cancer; NLR, neutrophil-to-lymphocyte ratio.

**Table 2:** Univariate analysis of predictors for disease-free survival.

Variables	HR (95% CI)	*p* Value
Age	1.02 (0.98–1.06)	0.402
Male gender	1.02 (0.45–2.33)	0.957
Current/former smoking	0.70 (0.26–1.86)	0.472
Upper/middle lobe	1.68 (0.65–4.31)	0.282
Surgical extent ^1^		
Wedge resection	2.47 (0.98–6.23)	0.057
Segmentectomy	0.76 (0.26–2.25)	0.618
Adjuvant treatment	1.30 (0.44–3.83)	0.636
Stage IB	1.69 (0.65–4.40)	0.283
Neutrophil percentage	1.04 (1.00–1.09)	0.055
Lymphocyte percentage	0.98 (0.94–1.02)	0.281
NLR	0.99 (0.91–1.07)	0.740
Histology		
Adenocarcinoma ^2^	1.21 (0.16–9.06)	0.851
Histological grade ^3^		
Well-differentiated	0.43 (0.10–1.93)	0.269
Moderately differentiated	0.45 (0.09–2.18)	0.321
IASLC grading ^4^		
Grade 1	0.31 (0.11–0.90)	0.031
Grade 2	0.36 (0.11–1.26)	0.110

^1^ lobectomy used as the reference; ^2^ squamous cell carcinoma used as the reference; ^3^ poorly differentiated used as the reference; ^4^ Grade 3 used as the reference; CI, confidence interval; HR, hazard ratio; IASLC, International Association for the Study of Lung Cancer; NLR, neutrophil-to-lymphocyte ratio.

**Table 3:** Univariate and multivariate analysis of predictors for overall survival.

Variables	Univariate HR (95% CI)	*p* Value	Multivariate HR (95% CI)	*p* Value
Age	1.10 (1.05–1.16)	<0.001		
Male gender	3.16 (1.24–8.06)	0.016		
Current/former smoking	2.43 (0.99–6.00)	0.054		
Upper/middle lobe	0.09 (0.02–0.38)	0.001	0.03 (0.00–0.33)	0.005
Surgical extent ^1^				
Wedge resection	1.51 (0.46–5.02)	0.499		
Segmentectomy	1.13 (0.35–3.66)	0.841		
Adjuvant treatment	2.30 (0.72–7.32)	0.160		
Stage IB	4.45 (1.80–10.97)	0.001	54.23 (5.24–561.15)	0.001
Neutrophil percentage	1.08 (1.02–1.14)	0.006		
Lymphocyte percentage	0.94 (0.89–0.99)	0.013		
NLR	1.02 (0.96–1.09)	0.483		
Histology ^2^				
Adenocarcinoma	4.06 (0.89–18.6)	0.071		
Histological grade ^3^				
Well-differentiated	0.11 (0.02–0.47)	0.003		
Moderately differentiated	0.04 (0.01–0.42)	0.007		
IASLC grading ^4^				
Grade 1	0.11 (0.02–0.48)	0.004	0.01 (0.00–0.13)	0.001
Grade 2	0.09 (0.01–0.83)	0.033	0.06 (0.00–0.77)	0.031

^1^ lobectomy used as the reference; ^2^ squamous cell carcinoma used as the reference; ^3^ poorly differentiated used as the reference; ^4^ Grade 3 used as the reference; CI, confidence interval; HR, hazard ratio; IASLC, International Association for the Study of Lung Cancer; NLR, neutrophil-to-lymphocyte ratio.

## Data Availability

The datasets used and analyzed in the current study are available from the corresponding author on reasonable request.
